# DIFFERENTIAL MRI ATROPHY PROFILES IN OLDER ADULTS WITHOUT DEMENTIA FROM AGING STUDIES IN THE US AND CZECH REPUBLIC

**DOI:** 10.1093/geroni/igad104.2547

**Published:** 2023-12-21

**Authors:** Monica Nelson, Brent Small, Zuzana Nedelska, Ondrej Lerch, Martin Vyhnalek, Ross Andel, Jakub Hort

**Affiliations:** University of Michigan, Ann Arbor, Michigan, United States; University of North Carolina-Chapel Hill, Chapel Hill, North Carolina, United States; Charles University and Motol University Hospital, Praha 5., Hlavni mesto Praha, Czech Republic; Charles University and Motol University Hospital, Praha 5., Hlavni mesto Praha, Czech Republic; Charles University and Motol University Hospital, Praha 5., Hlavni mesto Praha, Czech Republic; Arizona State University, Tempe, Arizona, United States; Charles University and Motol University Hospital, Praha 5., Hlavni mesto Praha, Czech Republic

## Abstract

Alzheimer’s disease is a progressive neurodegenerative disorder with extensive neuropathological heterogeneity. We aimed to identify distinct latent subgroups of neuropathology and differentiate them using sociodemographic, contextual, and cognitive factors. Participants without dementia from the Alzheimer’s Disease Neuroimaging Initiative (ADNI; n=1703) and the Czech Brain Aging Study (CBAS; n=385) were included. Latent profile analysis was used to detect profiles using 10 volumetric structural magnetic resonance imaging regions of interest derived using Freesurfer algorithm (hippocampus, middle temporal, superior temporal, precuneus, anterior cingulate, medial orbitofrontal, pericalcarine, precentral, lingual, and caudate regions). Four profiles emerged in ADNI: Minimal Atrophy (n=192), Mild Atrophy (n=691), Moderate Atrophy (n=567), Severe Atrophy (n=253). Two profiles emerged in CBAS: Mild Atrophy (n=208) and Severe Atrophy (n=177). For all but the caudate region, there were significant pairwise differences in volumetry between each profile. Further, Minimal Atrophy was the youngest, had the highest proportion of participants with normal cognition, had the lowest progression to dementia, and the best cognition compared to all other groups – the remaining pairwise comparisons suggested that participants were older, more likely to have mild cognitive impairment, progress to dementia, and have poorer cognition, transitioning from Mild to Moderate to Severe Atrophy. Findings were consistent in CBAS. In ADNI, Minimal and Mild Atrophy had higher education and premorbid abilities compared to Moderate and Severe Atrophy. In CBAS, Mild Atrophy had better premorbid abilities compared to Severe Atrophy. Biological heterogeneity among older adults without dementia should be considered to understand who may be at risk of cognitive decline.

